# Basal cell carcinoma of the scalp shows distinct features from the face in Asians

**DOI:** 10.1038/s41598-022-14533-y

**Published:** 2022-06-17

**Authors:** Minsu Kim, Jee-Woo Kim, Jung-Won Shin, Jung-Im Na, Chang-Hun Huh

**Affiliations:** Department of Dermatology, Seoul National University Bundang Hospital, 82 Gumi-Ro 173 Beon-Gil, Seongnam, 13620 Gyeonggi Korea

**Keywords:** Basal cell carcinoma, Basal cell carcinoma

## Abstract

Basal cell carcinoma (BCC) affecting different sites has been reported to have different clinicopathological features. In previous studies, the scalp was commonly classified to the head and neck region. However, the scalp has distinct characteristics from those of other parts of the skin. We retrospectively reviewed the medical records of patients who underwent surgical treatment for BCC. A total of 734 lesions were examined, and 13.2% originated from the scalp. The nodular type was the most common histologic subtype; however, the proportion of the superficial type was significantly higher than that of facial BCC (p < 0.001). Compared with facial BCC, younger age (p = 0.046) and larger tumor size (p < 0.001) were observed in scalp BCC. These characteristics were similar to those of truncal BCC in that they demonstrated a higher proportion of the superficial type (p < 0.001), younger age (p = 0.001), and larger tumor diameter (p < 0.001) compared with BCC in the head and neck region. Scalp BCC and truncal BCC were not significantly different in terms of age (p = 0.052) and tumor size (p = 0.230). In conclusion, despite the anatomical proximity, features of scalp BCC were similar to those of truncal lesions compared with facial lesions. Scalp BCC might be a separate entity from facial BCC.

## Introduction

Non-melanoma skin cancer including basal cell carcinoma (BCC) is the most commonly diagnosed cancer worldwide and the incidence of BCC has increased in both Western countries and Asia including Korea^1,2^. Although metastases of BCC are rare, local destruction in aggressive cases could lead to cosmetic and functional morbidities^3^. As most BCCs develop in the head and neck (H&N) region and their incidence is higher in the fair-skinned population, ultraviolet radiation (UVR) is considered to be an important factor in the pathogenesis of BCC^4^. However, some BCCs arise in sun-protected areas, which implies the presence of risk factors other than UVR affecting the development of BCC^5^. Several studies have revealed that clinical characteristics and histologic subtypes are different between H&N BCC and truncal BCC and claimed that BCC at different anatomic sites might be different entities^6,7^.

The scalp has been classified to the H&N region in most previous studies due to its close anatomical relationship. However, the features of the scalp are different from the skin of other areas in that it is composed of multiple layers and has a high density of pilosebaceous units^8,9^. Moreover, as the scalp is not directly exposed to UVR because of hair, scalp BCC might have different etiological factors from H&N lesions. For cutaneous melanoma, scalp lesions showed a significantly worse prognosis than facial lesions^10^. However, for BCC, although several studies have described the characteristics of scalp BCC, there are still lack of studies identifying features of scalp BCC by comparing them with BCC at other sites. Thus, this study aimed to investigate and compare the characteristics of scalp BCC with tumors at other sites.

## Methods

### Subjects

Retrospective review of the medical records of patients who underwent surgical treatment for BCC at the Seoul National University Bundang Hospital between January 1, 2010 and December 31, 2020 was performed. Histopathology of final excised specimens were also reviewed. The first two lesions were included in the analysis per patient as in a previous study^3^. If the second and third lesions could not be distinguished because more than two lesions were excised simultaneously, they were all included in the analysis. The exclusion criteria were recurrent tumors, tumors associated with genetic syndromes, and patients with risk factors for BCC, including a previous history of radiotherapy or an immunosuppressive state.

The collected data included sex, age, location of the lesions, and pathology reports, including the histologic subtype, tumor size, and invasion depth from the dermoepidermal junction. Anatomical location was first divided into H&N, trunk, extremities, and genitalia, similar to previous studies^3,5–7^. Then, the H&N region was further divided into the scalp, face, and neck, and facial lesions were reclassified according to the esthetic units suggested by Gonzalez-Ulloa and modified with reference to Oda T et al. as follows: frontal, nasal, orbital, cheek, labial, mental, auricular, and postauricular^11,12^. The histologic subtypes were classified according to the criteria arranged by Cameron et al.^1^.

According to the National Comprehensive Cancer Network (NCCN) Guidelines, tumors larger than 10 mm in the scalp, forehead, cheek, and neck were classified as high-risk BCC, while BCC in the other facial units were classified as high-risk BCC regardless of the size^13^. For the histologic subtype, infiltrative, morpheaform, micronodular, and metatypical types were considered as high-risk BCC^13^.

### Statistical analysis

Continuous and categorical variables were compared using the Mann–Whitney test or the Kruskal–Wallis test and chi-square test, respectively. Bonferroni correction was used to adjust for statistical significance in multiple comparisons. Multiple logistic regression was performed to investigate the association between the anatomical location and histologic subtype after adjustment for age and sex. SPSS 25.0 (IBM Corp., Armonk, NY, US) was used for statistical analysis, and it was interpreted as statistically significant when p-value was lower than 0.05.

### Ethics approval

The study was approved by the Seoul National University Bundang Hospital Institutional Review Board (IRB no. B-2102–666-104) and need of the informed consent was waived by ethics committee of Seoul National University Bundang Hospital Institutional Review Board. The study was conducted in compliance with the principles of the Declaration of Helsinki.

## Results

### Demographics

Among a total of 772 excised lesions from 717 patients, 734 lesions from 699 patients were included in the analysis (Table 1). All patients were Koreans. The mean age was 69.7 ± 12.6 years (range: 19–95 years) and 45.4% of the patients were males. A total of 90.2% of the lesions were located in the H&N. They were all treated with wide local excision.

**Table 1:** Characteristics of patients with basal cell carcinoma.

	H&N				Extra-H&N				p-value^b^	Total (n = 734)
	Face (n = 565)	Scalp (n = 97)	Total (n = 662)	p-value^a^	Trunk (n = 51)	Extremities (n = 17)	Genitalia (n = 4)	Total (n = 72)		
Sex^c^	269 (47.6)	26 (26.8)	295 (44.6)	< 0.001*	26 (51.0)	12 (70.6)	0 (0.0)	38 (52.8)	0.184	333 (45.4)
Age (years)	70.6 ± 12.0	67.9 ± 13.5	70.2 ± 12.2	0.046*	63.2 ± 14.7	68.1 ± 14.5	64.5 ± 17.4	64.5 ± 14.7	0.003*	69.7 ± 12.6
Tumor size (cm)	0.8 ± 0.5	1.2 ± 0.8	0.9 ± 0.6	< 0.001*	1.4 ± 1.0	1.0 ± 0.5	0.9 ± 0.6	1.3 ± 0.9	< 0.001*	0.9 ± 0.6
Invasion depth (mm)	2.0 ± 1.4	2.0 ± 1.9	2.0 ± 1.5	0.785	2.4 ± 4.4	1.5 ± 0.9	1.8 ± 1.7	2.1 ± 3.8	0.077	2.0 ± 1.8
Subcutaneous tissue involvement	96 (17.0)	9 (9.3)	105 (15.9)	0.055	4 (7.8)	0 (0.0)	0 (0.0)	4 (5.6)	0.020*	109 (14.9)
Lymphovascular invasion	7 (1.2)	0 (0.0)	7 (1.1)	0.602	1 (0.2)	0 (0.0)	0 (0.0)	1 (1.4)	0.564	8 (1.1)
Perineural invasion	5 (0.9)	0 (0.0)	5 (0.8)	1.000	0 (0.0)	0 (0.0)	0 (0.0)	0 (0.0)	1.000	5 (0.7)
**Histologic subtype**										
Nodular	407 (72.0)	63 (64.9)	470 (71.0)	0.155	23 (45.1)	9 (52.9)	3 (75.0)	35 (48.6)	< 0.001*	505 (68.8)
Superficial	54 (9.6)	25 (25.8)	79 (11.9)	< 0.001*	21 (41.2)	5 (29.4)	0 (0.0)	26 (36.1)	< 0.001*	105 (14.3)
Infiltrative	50 (8.8)	1 (1.0)	51 (7.7)	0.008*	3 (5.9)	0 (0.0)	1 (25.0)	4 (5.6)	0.511	55 (7.5)
Mixed	6 (1.1)	2 (2.1)	8 (1.2)	0.332	2 (3.9)	1 (5.9)	0 (0.0)	3 (4.2)	0.084	11 (1.5)

Data are presented as mean ± standard deviation or number (%). p-values were calculated using the Mann–Whitney test and chi-square test for continuous and categorical variables, respectively.

*H&N* head and neck region.

^a^Comparison between face and scalp lesions.

^b^Comparison between H&N and extra-H&N lesions.

^c^Percentage of males.

*p < 0.05.

The nasal unit (36.7%) was the most frequently involved unit in the H&N region, followed by the cheek unit (17.4%), orbital unit (15.4%), and scalp (14.7%). Among the extra-H&N lesions, 70.8% were truncal lesions and 23.6% of the lesions were located in the extremities.

**Figure 1:**
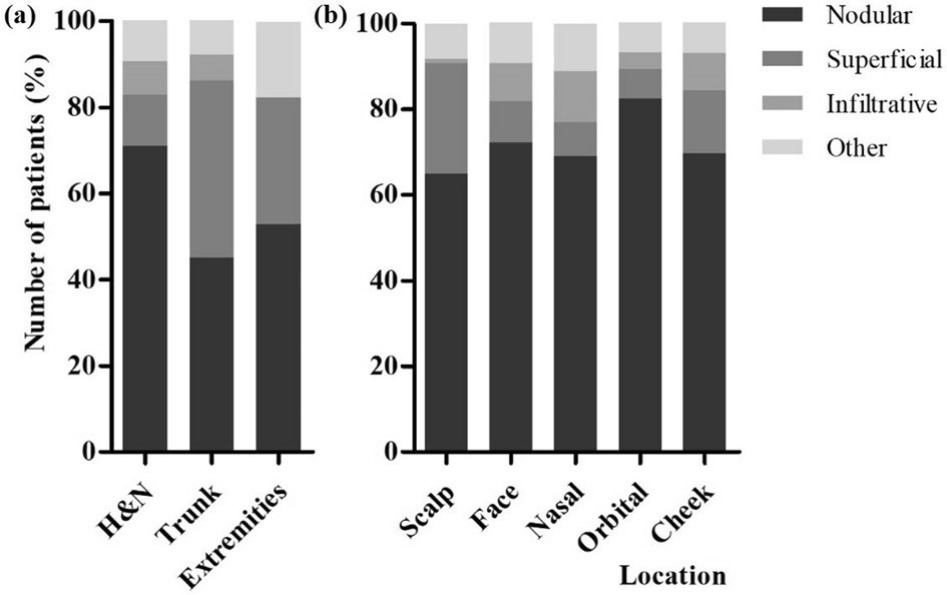
Proportion of the histologic subtype of basal cell carcinoma according to the location (**a**) divided into H&N, trunk, extremities, (**b**) among H&N lesions. *H&N* head and neck.

As every excised specimen contained residual tumor, only the final excised specimens, not the initial biopsies, were included in the analysis. Regarding the histologic subtype, the nodular type (68.8%) was the most common subtype, followed by the superficial type (14.3%) and infiltrative type (7.5%) (Fig. 1). Other types were excluded from further analysis due to their low numbers.

### Comparison between H&N and extra-H&N BCC

Clinical characteristics were compared between the H&N, trunk, and extremities. Genital lesions were excluded from further analysis because of their low numbers. Sex was not associated with the tumor location (p = 0.184). Patients with truncal lesions were younger (63.2 ± 14.7 years) than those with H&N lesions (70.2 ± 12.2 years, p = 0.001). The tumor size was larger in truncal lesions (1.4 ± 1.0 cm) than in H&N lesions (0.9 ± 0.6 cm, p < 0.001). There was no difference in the invasion depth according to the location (p = 0.077).

Extra-H&N location was strongly associated with the superficial type compared with the nodular type (odds ratio [OR] = 4.003, p < 0.001) or infiltrative type (OR = 3.703, p = 0.023) after adjustment for sex and age. Furthermore, the trunk favored the superficial type than the nodular type (OR = 4.858, p < 0.001) or infiltrative type (OR = 4.032, p = 0.032).

### Comparison between scalp and facial BCC

Scalp BCC was identified in 13.2% of all BCC cases. Females comprised 73.2% of the cases of scalp BCC, and its female predominance was significant compared with other facial units (p < 0.001). Age and tumor size did not differ according to sex among scalp lesions (p = 0.568 and p = 0.701, respectively).

Although the nodular type was the most common histologic subtype, the scalp demonstrated a higher percentage of the superficial type (25.8%) than other facial units (9.6%, p < 0.001). This preference of the superficial type was statistically significant after adjustment for age and sex compared with the nodular type (OR = 2.751, p < 0.001) or infiltrative type (OR = 24.390, p = 0.002).

Younger age was associated with scalp lesions (67.9 ± 13.5 years) compared with the face (70.6 ± 12.0 years, p = 0.046). Tumor diameter was larger in the scalp (1.2 ± 0.8 cm) than in the face (0.8 ± 0.5 cm, p < 0.001). The invasion depth was similar between the two groups (p = 0.785). When compared with the trunk, age and the tumor size of scalp lesions were not significantly different (p = 0.052 and p = 0.230, respectively).

According to the NCCN Guidelines, 55.7% of scalp BCCs and 83.0% of facial BCCs were high-risk BCC in terms of their location and size. For the histologic subtype, 6.2% of scalp BCCs and 15.6% of facial BCCs were classified as high-risk BCC. The rate of subcutaneous tissue involvement was lower in the scalp than in the face without statistical significance (p = 0.055). No lymphovascular invasion or perineural invasion was noted in the scalp lesions.

## Discussion

Previous studies have claimed that BCC affecting different sites demonstrates different clinicopathological characteristics^3,5,6^. Considering that scalp is covered with hair with a high density of hair follicles, and since BCC is thought to originate from follicular basal cells, scalp lesions may have characteristics different from facial BCC^8^. Few studies have reported the features of scalp BCC; however, a study comparing the characteristics of scalp BCC with BCC at other sites with a large number of patients is required^14,15^.

Scalp BCC accounted for 13.2% of the total BCCs, which was within the previously reported range^8^. Female predominance was prominent in scalp BCC in our study. This result is different from most previous studies that reported a higher prevalence of scalp BCC among males^14–17^. Since there are differences in the clinical and histopathological characteristics of BCC between Caucasians and Asians, this could be due to ethnic differences, as previous studies were conducted in Caucasians^18^. Some studies explained the male predominance of scalp BCC with a higher incidence of androgenetic alopecia among males^14,19^. According to the recent study conducted by Chlebicka et al., skin lesions on the scalp including BCC mainly occurred in the frontal, temporal, and apex area of the scalp in Caucasian population, which are the commonly affected areas by androgenetic alopecia^20^. Thus, a lower prevalence of androgenetic alopecia among Korean males compared with Caucasians might also partially explain this result^21^.

The nodular type was the most common histologic subtype in scalp BCC, which was consistent with previous reports^14,15^. However, we observed that the proportion of superficial BCC was higher than that of facial BCC, and the difference was significant after adjusting for age and sex. This pattern was similar to that of truncal BCC. As with previous studies, we confirmed the preference of the superficial type in the trunk^3,5,6,22^. Pelucchi et al. revealed that occupational sun exposure was associated with the nodular type and facial lesions but not associated with the superficial type or truncal lesions^7^. Thus, the tendency to develop a specific subtype at a specific location might be due to shared risk factors. As both the scalp and trunk are sun-protected areas, this might explain the preference of the superficial type in both areas.

The similarity between scalp BCC and truncal BCC was also noted in terms of age and tumor size. Although the patients were younger in these groups, their tumor size was larger than that of facial BCC. This can be interpreted in two ways. First, as the scalp and trunk are less visible than the face, this could be the result of delayed recognition. This implies that their true onset was even earlier than that of facial BCC. Second, they could have a faster growth rate than facial BCC. This strengthens our hypothesis that scalp and truncal BCC have characteristics different from facial BCC. BCC occurring on the scalp and trunk were less frequently accompanied by solar elastosis than in the case of the face, and the degree was weaker^23^. Thus, earlier onset in the non-exposed area implies the presence of factors other than UVR involved in the occurrence of BCC. Genetic predisposition could be an explanation as genetic polymorphisms in glutathione S-transferase (GSTT1) and cytochrome P450 (CYP1A1) were reported to be associated with multiple truncal BCC^24^. Moreover, it has recently been revealed that NOTCH1 mutations are significantly associated with superficial subtype and truncal locations of BCC respectively^25^. It could be assumed that certain genetic alterations affects the early development of BCC on non-exposed areas than patients without such alterations who may require the time for BCC to develop after the accumulation of UVR-related changes leading to carcinogenesis in the face.

It cannot be concluded that scalp BCC and truncal BCC are the same entity as they had different sex distributions in our study and different dermoscopic features were observed between them^26^. However, it is interesting that despite the anatomical proximity to the face, scalp BCC showed characteristics similar to truncal BCC rather than facial BCC. This result suggests that scalp BCC might be a distinct entity from facial BCC. To the best of our knowledge, this is the first study to report on certain relationships.

Our study has a limitation that due to its retrospective nature, we could not assess the several clinical information including exact location of the scalp BCC or history of alopecia. In particular, regarding the prognosis of scalp BCC, Castanheira et al. claimed that scalp BCC have been reported as an aggressive tumor^8^. Unfortunately, in current study, data on recurrence rate or morbidity were lacking. Thus, although our data showed that the proportion of high-risk BCC was lower in scalp BCC than that of facial BCC, it was unable to clearly discuss the actual prognosis of scalp BCC. However, our study emphasizes that the scalp requires a thorough physical examination as an earlier onset of BCC and hair coverage could result in late detection of lesions, and larger tumor size would cause difficulty in surgery due to the tightness and lack of surrounding reservoir tissue in the scalp^27^. Moreover, our study is meaningful in that we analyzed the characteristics of a large number of scalp BCC patients in the non-Caucasian population.

In conclusion, scalp BCC seems to be a separate entity that has characteristics different from facial BCC and is rather similar to truncal BCC. However, as our study was performed at a single institution, we expect further studies to verify the distinct characteristics of scalp BCC in other patient groups.

## Data Availability

The datasets used and analyzed during the current study available from the corresponding author on reasonable request.
